# Brain transcriptome changes in the aging *Drosophila melanogaster* accompany olfactory memory performance deficits

**DOI:** 10.1371/journal.pone.0209405

**Published:** 2018-12-21

**Authors:** Rodrigo Pacifico, Courtney M. MacMullen, Erica Walkinshaw, Xiaofan Zhang, Ronald L. Davis

**Affiliations:** Department of Neuroscience, The Scripps Research Institute-Florida, Jupiter, Florida, United States of America; Biomedical Sciences Research Center Alexander Fleming, GREECE

## Abstract

Cognitive decline is a common occurrence of the natural aging process in animals and studying age-related changes in gene expression in the brain might shed light on disrupted molecular pathways that play a role in this decline. The fruit fly is a useful neurobiological model for studying aging due to its short generational time and relatively small brain size. We investigated age-dependent changes in the Drosophila melanogaster whole-brain transcriptome by comparing 5-, 20-, 30- and 40-day-old flies of both sexes. We used RNA-Sequencing of dissected brain samples followed by differential expression, temporal clustering, co-expression network and gene ontology enrichment analyses. We found an overall decline in expression of genes from the mitochondrial oxidative phosphorylation pathway that occurred as part of aging. We also detected, in females, a pattern of continuously declining expression for many neuronal function genes, which was unexpectedly reversed later in life. This group of genes was highly enriched in memory-impairing genes previously identified through an RNAi screen. We also identified deficits in short-term olfactory memory performance in older flies of both sexes, some of which matched the timing of certain changes in the brain transcriptome. Our study provides the first transcriptome profile of aging brains from fruit flies of both sexes, and it will serve as an important resource for those who study aging and cognitive decline in this model.

## Introduction

The natural process of aging in animals entails changes at the behavioral, anatomical, cellular and molecular levels. Global transcriptome studies offer an unbiased method to assay changes in gene expression associated with aging and may provide insights into pathways that play a role in that process. However, variable genetic background, diverse environments, long lifespans and limited access to the tissue of interest are some of the major difficulties faced when performing such studies directly in humans, particularly in the brain. In this regard, animal models can provide an important complement for investigating the molecular changes underlying the cognitive deficits that occur with aging.

Transcriptome profiling studies of the aging brain have been performed in various model organisms, including mice [1], zebrafish [2] and non-human primates [3]. Some of these studies have revealed common themes in age-related gene expression changes, such as upregulation of stress response and inflammation/immune response genes, as well as the downregulation of oxidative metabolism and neuronal/synaptic function genes [4]. Many of the transcriptome changes observed in aging brains from model organisms have also been documented in gene profiling studies of postmortem human brains [5, 6], validating the usefulness of these models.

The fruit fly is a valuable model organism in biomedical research, particularly due to its tractability to genetic manipulation, short generational time and ease of maintenance. As a model in neurobiology, the fly brain offers the advantage of being relatively small and yet capable of producing complex behaviors. Studies of the fly brain have provided important insights into human neurological disorders such as Alzheimer’s [7] and Parkinson’s disease [8, 9]. The fly brain is also a useful model for studying the normal aging process and its associated cognitive changes, as studies have shown disturbances in memory [10–12], sleep [13–15], locomotion [16–18] and other behaviors [19] in aging flies. Age-related memory impairment in the fruit fly can be suppressed pharmacologically [20], thus also making it a useful model in translational research for screening compounds that enhance memory or slow its decline with age. Despite the value of *Drosophila* as a model organism in studying the neurobiology of aging, few transcriptome studies of the aging fruit fly brain have been reported, and they were either restricted to males [21] or relied on RNA extracted from heads [22, 23], which contain many tissue types, rather than dissected brains. In addition, these older studies were performed using microarray technology, which is more susceptible to experimental variability and background noise than RNA sequencing approaches [24, 25], and is limited in its ability to detect novel transcripts. Recently, a single-cell RNA-Seq study of the aging fruit fly brain was published [26], but samples from both sexes were pooled in that study, precluding the analysis of age-related gene expression changes in each separate sex.

Here we provide the transcriptome profiling of whole-brains from the fruit fly *Drosophila melanogaster*, derived from both sexes and collected at different ages during the life of the fly. We show age-related changes in expression of genes in pathways previously identified in aging studies of the human brain and model organisms. Furthermore, we identify a co-expression module that is highly enriched in genes known to affect learning and memory in flies. We also show age-dependent memory performance deficits in the same strain of flies, some of which parallel certain changes observed in the brain transcriptome.

## Material and methods

### Fly stocks and husbandry

*Drosophila melanogaster* Canton-S (CS) fly stocks were maintained at 25°C and 70% humidity under a 12:12-hour light/dark cycle. Flies were collected twice daily, to ensure age, and separated by sex. Flies were flipped to fresh food 1–2 times per week until the desired age was reached. For survival curve analysis, 600 male flies and 600 female flies were collected after eclosion. Every 3 days, the surviving flies were transferred to fresh vials and deaths were scored.

### RNA-sequencing

CS flies were collected in vials containing either 50 male flies or 50 female flies and were aged to 5, 20, 30 or 40 days post-eclosion. At each of the time points, whole brains from 18 flies were dissected in RNALater solution (Ambion, Foster City, CA). Triplicate biological samples were collected for both males and females at each time point, although only two of the three samples were used for the 30-day-old male group, since the third sample of this group was later found to have female contaminants. RNA was isolated from each sample using the Qiagen RNeasy Plus Micro Kit (Qiagen, Venlo, Netherlands) and treated with DNaseI using the TURBO DNA-free kit (Life Technologies, Carlsbad, CA), according to the manufacturers’ instructions. Twenty-five nanograms of RNA from each sample was used to generate cDNA libraries using the Ovation Universal RNA Seq V2 Kit for *Drosophila* (Nugen, San Carlos, CA) according to manufacturer’s instructions. Libraries were multiplexed so that each sample would generate approximately 30 million paired-end reads and run on a NextSeq 500 sequencer (Illumina, San Diego, CA).

### Raw sequencing data processing

Paired-end sequencing reads were trimmed to remove adapter sequences and low-quality base reads using Trimmomatic (v0.35) [27] with standard settings. Samples were aligned against the full *D*. *melanogaster* genome using the splice-aware STAR aligner [28]. Finally, the HTSeq-count script [29] was used in “union” mode to count reads that mapped unambiguously to annotated features on the assembled genome. Reference genome and genome annotation files were obtained from the *D*. *melanogaster* genome browser, assembly dm6, hosted on the University of California-Santa Cruz (UCSC) server. A file with read counts per gene for all samples (raw data, not normalized) is included in the Supporting Information.

### Differential expression analysis

Analysis of differentially expressed genes was performed using the R package DESeq2 [30]. Library sample sizes were normalized using the default parameters. The effect of age on the expression of each gene was tested by comparing the full model and the reduced model (i.e. without “age” as a factor) through the likelihood ratio test (LRT). Differential expression between two age points was then tested using the Wald’s test of log-fold changes. In each of these two tests, outlier genes were flagged using the default Cook’s distance cutoff, and genes with very low read counts were automatically removed through the default independent filtering method. All genes that remained after filtering are referred to as “detectable” in all further analyses. P-values were adjusted for multiple testing by the false-discovery rate (FDR) Benjamini-Hochberg method. The regularized logarithmic transform was used to convert discrete read counts into continuous r-log values that were used in the temporal clustering and co-expression network analyses.

### Fuzzy c-means clustering

Temporal clustering was performed using the R package Mfuzz [31]. For each age group, r-log expression values for each DE gene were averaged across samples. Expression values for all genes were then standardized by subtracting the mean value across ages and dividing by the standard deviation. The fuzzifier parameter *m* and the number of clusters *c* were estimated using a previously described method [32]. The α = 0.4 threshold was used for identifying the core genes in each cluster. Only the core genes were plotted in the temporal profiles and used when calculating enrichment in gene ontology terms.

### Weighted gene co-expression network analysis

Weighted gene co-expression network analysis [33] was performed using the WGCNA package (v1.51) in R; the r-log values from all detectable genes in each dataset were used. The soft-thresholding power used for both datasets was 7; this was determined using the scale-free topology criterion. For each dataset, a network was built using the blockwiseModules function with the following parameters: networkType = "unsigned", TOMType = "signed", corType = "bicor", minModuleSize = 40, mergeCutHeight = 0.2, minKMEtoStay = 0.8. The maxBlockSize parameter was set to the total number of genes in order to ensure calculations were done in a single block. The association of each module with age was tested by calculating the effect of age on eigengene variation with ANOVA. Eigengene plots were generated by fitting eigengene values onto a curve smoothed by the LOESS method. The iGraph R package [34] was used to generate module plots showing the top 200 links (based on the adjacency matrix of absolute value biweight midcorrelations for genes in that module).

### Functional enrichment analyses

Enrichment of gene ontology (GO) terms from the “Biological Process” class was calculated in a given gene set (DE genes set, temporal cluster or co-expression module) using the GORILLA tool [35]. In each analysis, all detectable genes in that dataset (see Differential expression analysis sub-section above) were used as background, and enrichment was calculated using the hypergeometric probability test. All GO terms significant after multiple testing correction (at a 5% false-discovery rate threshold, Benjamini-Hochberg method) were submitted to the REVIGO tool along with p-values for generating non-redundant lists containing the most representative terms; the “allowed similarity” parameter was set to “small” (0.5). Enrichment in genes that affect memory was calculated using hits from a previous RNAi screen [36]; the enrichment was calculated separately for memory-enhancing and memory-impairing hits (respectively defined as genes that either increased or decreased performance when knocked down in an olfactory learning aversive conditioning task within that study). Similar to the GO enrichment analysis, all detectable genes were used as background in the hypergeometric probability test.

### Behavior

We used 5, 20, 30 or 40-day-old flies for behavioral experiments. Flies were collected 24 hours before experiments and were transferred to fresh food vials 15 minutes before conditioning for equilibration to the experimental room conditions of 25°C and 70% humidity. Experiments were performed in a dark room under red-filtered light. Standard aversive olfactory conditioning experiments were performed as described [37]. Each experimental group of 60 flies was loaded into a training tube where they received the following sequence of stimuli: 30 seconds of air, 1 minute of an odor paired with 12 pulses of 90 V electric shock (conditioned stimulus, CS^+^), 30 seconds of air, 1 minute of a second odor with no electric shock (CS^−^), and a final 30 seconds of air. For conditioning odors, we bubbled fresh air through 3-octanol (OCT) or benzaldehyde (BEN) at concentrations in mineral oil that provided for optimal balance in the half performance index (PI) between odors. Optimal odor concentrations varied across time but were generally between 0.065% and 0.075% for benzaldehyde and 0.15% for 3-octanol. After conditioning, flies were either returned to a food vial to be tested at the 1-hour or 3-hour time point, or transferred immediately to the T-maze for 3-minute memory testing. Flies were allowed a 1-minute resting period followed by a 2-minute decision period to choose between a *T*-maze arm exposing the flies to the CS^+^ odor and an arm exposing the flies to the CS^−^ odor. For all experiments, two groups were trained and tested simultaneously. One group was trained with OCT as the conditioned stimulus paired with electric shock (CS^+^) and BEN unpaired with electric shock (CS^−^), while the other group was trained with BEN as CS^+^ and OCT as CS^−^. Each group tested provides a half PI: Half PI = [(no. flies in CS^−^ arm) − (no. flies in CS^+^ arm)]/(no. flies in both arms). A final PI was calculated by averaging the two half PIs. Since the two groups were trained to opposite CS^+^/CS^−^ odor pairs, this method balances out naive odor biases.

## Results

### Differential gene expression in the aging fly brain

RNA-Seq was performed using isolated brains from male and female adult Canton-S flies of 5, 20, 30, and 40 days of age. Principal component analyses of sequencing data revealed some separation of samples according to age along the axes of the first two components, irrespective of sex (S1 Fig). However, this analysis also indicated a higher variance in the female data—therefore, we analyzed data from each sex separately in this paper. Differential expression analysis was first accomplished using the likelihood-ratio-test (LRT) in DESeq2, which identifies genes whose expression changes significantly when the independent variable (age) is included in the full model, in comparison to the reduced model in which the “age” variable is absent. Unsurprisingly, there were a large number of genes that changed in expression as a function of age (Table 1; see S1 Table for the full list of genes). In order to identify genes that are differentially expressed (DE) at different ages in older flies (20, 30 and 40 days of age) in comparison to young flies (5 days old), we used the Wald’s test, which tests the probability of finding the same (or higher) log-fold change in expression between two groups by chance. Only genes identified as statistically significant in LRT and Wald’s test were considered to be differentially expressed in the pairwise comparisons. Using this dual filtering approach, the largest number of DE genes (1521) was found in the brain of 30-day-old female flies, and the smallest number of DE genes (66) was detected in the brain of 30-day-old male flies. However, the latter is likely in part due to the smaller sample size of this group (only two 30-day-old male samples were used; see Materials and Methods).

**Table 1:** Number of DE genes in each pairwise age comparison.

Sex	Comparison	All	Up	Down
Male	All ages (LRT)	1076	-	-
	20d vs. 5d	217	105	112
	30d vs. 5d	66	28	38
	40d vs. 5d	808	407	401
Female	All ages (LRT)	3271	-	-
	20d vs. 5d	913	413	500
	30d vs. 5d	1521	642	879
	40d vs. 5d	1496	730	766

Number reflects genes that were significant after multiple-testing correction at 5% FDR in both the Wald’s test and the LRT.

**Fig 1:**
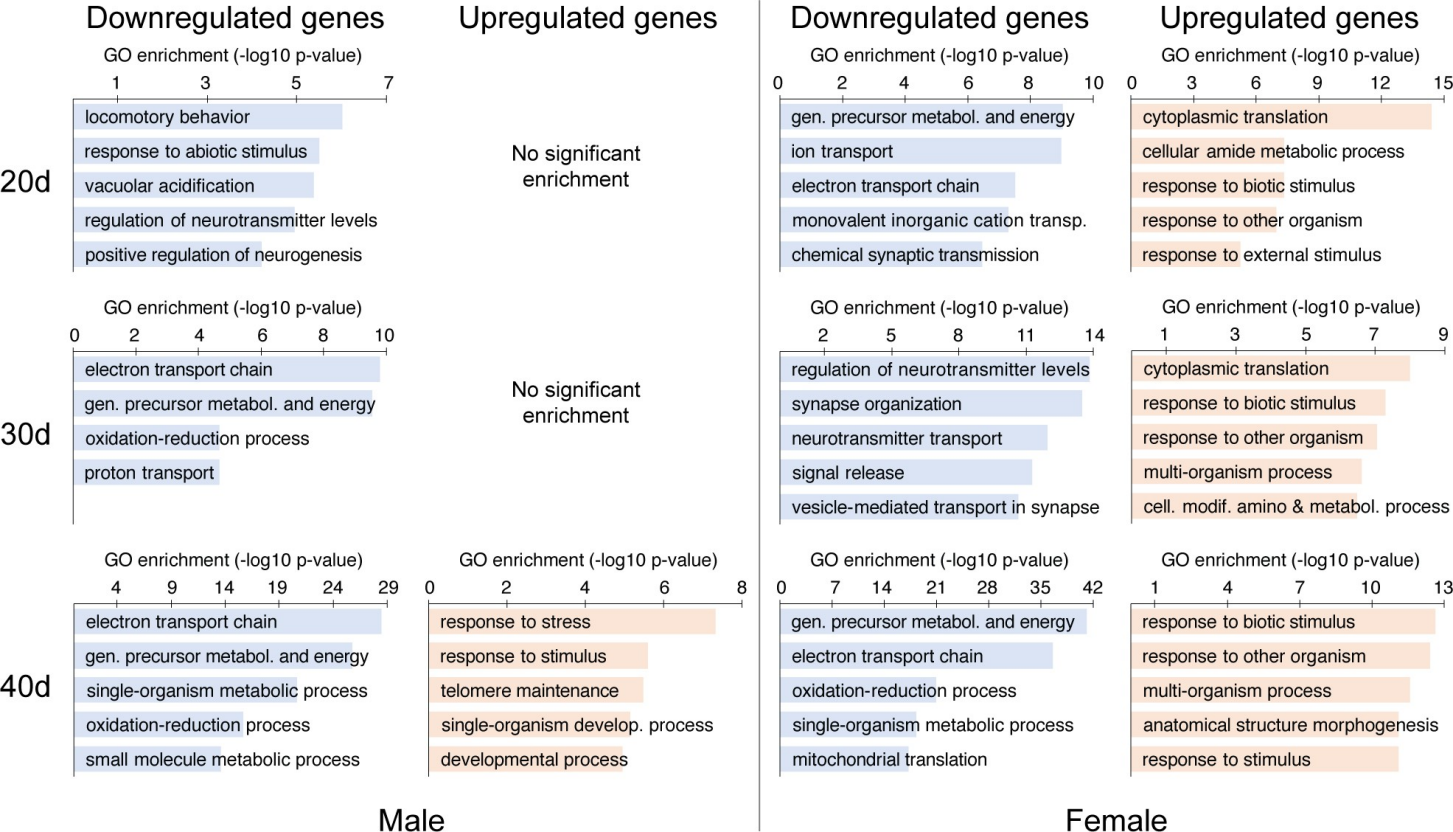
GO term enrichment for the sets of genes differentially expressed in older flies at different ages (20, 30 and 40 days old) in comparison to young flies (5 days old). In each group, the top terms (up to 5, listed by decreasing significance) are shown. The full lists can be found in S2 Table.

Gene ontology enrichment analysis, which provides a broader view of functional classes that are overrepresented in a given gene set, was performed for DE genes at each pairwise comparison, in each sex, using the “Biological Processes” category. This analysis was also carried out for upregulated and downregulated genes separately, in order to distinguish whether the identified processes were amplified or diminished with age. Enrichment results were filtered to remove redundant GO terms and generate concise, more meaningful lists (see Material and Methods). The top 5 terms in each pairwise comparison, separated by sex and direction, are shown in Fig 1 (full lists are available in S2 Table). At every age in females, and in 30- and 40-day-old males, there was a decrease in expression of energy metabolism genes in the brain, particularly genes from the mitochondrial electron transport chain, when compared to young flies. In the female samples, there was also an age-dependent increase in genes involved in responding to external challenges, including stress response genes. In males, this enrichment was only detected in the oldest group.

### Clustering differentially expressed genes by temporal profiles

In the analyses above, we identified genes that change expression later in life, using the young fly brain as a baseline. However, this does not distinguish genes that change continuously through life versus genes that only change between adjacent age points. For instance, a gene differentially expressed at 40 days of age may have changed continuously with aging or, alternatively, it may have changed in expression during a shorter period of time, remaining stable otherwise. Additionally, genes with a U-shaped temporal profile would not be detected in a pairwise comparison between more distal age points. Since the large number of DE genes makes examination of individual genes impractical, we used the fuzzy c-means (FCM) clustering method to group DE genes according to their temporal profiles. In other words, this method clusters genes whose expression levels across time change in a similar fashion. We used all genes identified as DE in the LRT test. The optimal number of clusters, determined using the minimum distance between cluster centroids approach, was 12 for the male dataset and 15 for the female dataset. Male and female clusters are henceforth abbreviated MC and FC, respectively (Table 2; see also S2 Fig).

**Table 2:** Clusters identified by fuzzy c-means method.

		Core	MEG enrichment			MIG enrichment		
Cluster	Genes	genes	Genes	p-value	q-value	Genes	p-value	q-value
*Male*								
MC1	65	14	0	1	1	0	1	1
MC2	102	36	0	1	1	0	1	1
MC3	82	33	0	1	1	0	1	1
MC4	106	48	0	1	1	0	1	1
MC5	77	25	0	1	1	0	1	1
MC6	70	12	0	1	1	0	1	1
MC7	91	29	0	1	1	2	0.380	1
MC8	117	46	0	1	1	3	0.346	1
MC9	92	24	0	1	1	0	1	1
MC10	97	36	0	1	1	2	0.489	1
MC11	87	41	0	1	1	3	0.283	1
MC12	90	23	0	1	1	2	0.279	1
*Female*								
FC1	234	76	0	1	1	5	0.264	0.659
FC2	234	76	0	1	1	4	0.457	0.685
FC3	223	60	0	1	1	0	1	1
FC4	168	45	0	1	1	3	0.336	0.685
FC5	202	53	0	1	1	3	0.436	0.685
FC6	235	59	1	0.188	1	2	0.756	0.946
FC7	222	55	0	1	1	2	0.721	0.946
FC8	174	40	0	1	1	1	0.845	0.975
FC9	232	48	0	1	1	5	0.066	0.329
FC10	207	47	0	1	1	4	0.164	0.496
FC11	238	82	3	0.003	0.044	14	1.72E-05	1.29E-04
FC12	237	66	0	1	1	0	1	1
FC13	207	64	0	1	1	5	0.165	0.496
FC14	201	33	0	1	1	2	0.447	0.685
FC15	257	98	1	0.292	1	19	6.87E-08	1.03E-06

MC and FC are male and female clusters, respectively. MEG, Memory-Enhancing Gene; MIG, Memory-Impairing Gene. Q-values are FDR-adjusted p-values.

**Fig 2:**
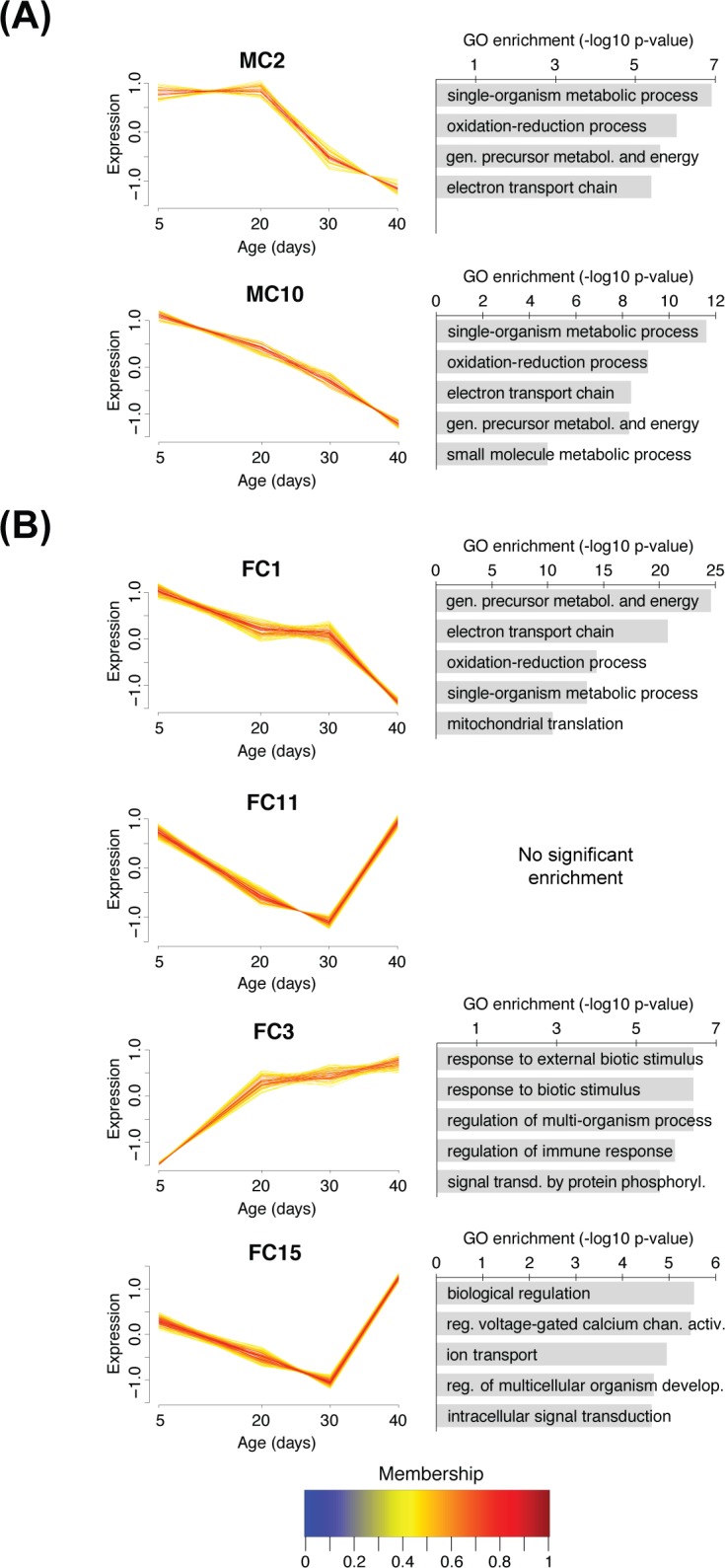
Clustering of DE genes by temporal profiles through the fuzzy c-means method. (A) Temporal profile and GO enrichment of two clusters from the male dataset. (B) Temporal profile and GO enrichment of four clusters from the female dataset. Not all clusters are shown. Individual lines represent expression of single genes averaged across samples, colored according to the cluster membership values shown in the color bar; only core genes (α ≥ 0.4) are plotted (see Material and Methods). For GO enrichment, the top terms (up to 5, listed by decreasing significance) are shown. The full lists can be found in S3 Table.

Some clusters provided informative findings based on their temporal profiles and functional enrichment (Fig 2 and S3 Table). One cluster in the female data (FC1) showed an overall decrease in expression with aging (Fig 2B); interestingly, the shifts in expression occurred mostly in the earlier (5 to 20 days) and later periods (30 to 40 days), whereas expression in the middle phase (20 to 30 days) was largely unchanged. This cluster was significantly enriched in GO terms related to metabolic oxidation-reduction processes, mirroring the findings of the enrichment analysis done when comparing downregulated genes in older vs. 5-day-old female flies. Two clusters in the male data (MC2 and MC10) showed a similar age-related decline in expression, and a similar enrichment in electron transport chain genes (Fig 2A). However, MC2 showed a delayed pattern where the decline in expression only began at 20 days of age, whereas MC10 showed a decline beginning earlier. Both clusters overlapped significantly with FC1 (p = 1.86 x 10^−7^ for MC2 and p = 8.43 x 10^−18^ for MC10; hypergeometric probability test). These results underscore a reduction in expression of components from the mitochondrial oxidative phosphorylation machinery in older flies of both sexes.

In order to gain further insight about the functional relevance of the gene clusters with respect to cognition, we tested their enrichment in genes that are known to affect memory performance. These genes were previously identified through an RNAi screen as genes that, when knocked down, either impair or enhance memory performance in the same aversive olfactory learning task described here [36]. Two clusters in the female data, FC11 and FC15, were significantly enriched in memory-impairing hits, while FC11 also showed a weak enrichment in memory-enhancing hits (Table 2). The FC15 cluster was enriched in GO terms related to cell signaling and ion channels. These clusters had an unexpected temporal profile, where expression declined roughly monotonically between 5 and 30 days of age, but then showed a sharp reversal of this decline between 30 and 40 days of age (Fig 2B).

### Gene co-expression network analysis

The previous clustering analysis was restricted to DE genes and grouped genes with similar temporal expression profiles. In order to obtain a snapshot of the entire aging brain transcriptome and its changes, we used the weighted gene co-expression network analysis to group genes with correlated expression patterns, irrespective of DE status. In other words, this method groups genes whose expression levels across samples behaves in concerted fashion (e.g. when gene A is elevated in a sample, so is gene B; changes in opposite directions are also possible, as long as they are consistent across samples). We built separate co-expression networks for the male and female datasets, using the same parameters (see Material and Methods). Genes in the male dataset were assigned to a total of 19 modules; genes in the female dataset were assigned to a total of 23 modules. We tested whether the overall expression of each module, summarized by its eigengene, varied significantly with respect to age. We found a large number of modules (5 in males and 13 in females) whose eigengenes were statistically associated with age (Table 3) through this ANOVA test.

**Table 3:** Modules identified by gene co-expression network analyses.

		Age		MEG enrich.			MIG enrich.		
Mod.	Gns.	p-value	q-value	Gns.	p-val.	q-val.	Gns.	p-value	q-value
*Male*									
MM1	157	0.586	0.619	1	0.423	1	7	0.571	0.996
MM2	48	0.180	0.298	1	0.154	0.980	0	1	1
MM3	52	0.233	0.316	0	1	1	1	0.910	0.996
MM4	41	0.195	0.298	0	1	1	3	0.283	0.996
MM5	41	0.203	0.298	0	1	1	3	0.283	0.996
MM6	363	0.035	0.094	1	0.723	1	11	0.943	0.996
MM7	283	0.003	0.035	1	0.631	1	11	0.740	0.996
MM8	220	0.007	0.035	1	0.539	1	15	0.074	0.705
MM9	68	0.011	0.043	0	1	1	2	0.820	0.996
MM10	217	0.026	0.082	0	1	1	14	0.115	0.731
MM11	1957	0.306	0.359	12	0.029	0.551	152	8.25E-13	1.57E-11
MM12	183	0.322	0.359	0	1	1	6	0.842	0.996
MM13	243	0.004	0.035	2	0.206	0.980	12	0.420	0.996
MM14	453	0.007	0.035	1	0.800	1	17	0.821	0.996
MM15	62	0.266	0.337	1	0.195	0.980	3	0.537	0.996
MM16	576	0.199	0.298	2	0.602	1	22	0.826	0.996
MM17	44	0.204	0.298	0	1	1	3	0.321	0.996
MM18	53	0.973	0.973	0	1	1	3	0.432	0.996
MM19	55	0.058	0.138	0	1	1	1	0.922	0.996
*Female*									
FM1	46	0.066	0.089	0	1	1	2	0.626	0.959
FM2	129	1.32E-05	1.52E-04	0	1	1	13	0.006	0.067
FM3	54	0.019	0.036	1	0.173	1	3	0.448	0.959
FM4	56	0.009	0.027	0	1	1	4	0.250	0.822
FM5	366	0.001	0.006	3	0.136	1	27	0.009	0.070
FM6	94	0.764	0.764	1	0.282	1	4	0.625	0.959
FM7	615	0.012	0.030	2	0.644	1	19	0.976	1.000
FM8	49	0.026	0.046	0	1	1	1	0.898	1.000
FM9	48	0.009	0.027	0	1	1	2	0.649	0.959
FM10	583	8.13E-07	1.87E-05	0	1	1	23	0.792	0.959
FM11	146	0.064	0.089	1	0.403	1	9	0.221	0.822
FM12	41	0.216	0.248	0	1	1	2	0.563	0.959
FM13	1242	0.013	0.031	11	0.003	0.063	129	1.48E-20	3.40E-19
FM14	101	0.005	0.022	1	0.300	1	8	0.089	0.510
FM15	178	0.001	0.005	0	1	1	7	0.706	0.959
FM16	41	0.043	0.066	0	1	1	3	0.286	0.822
FM17	48	0.038	0.063	0	1	1	2	0.649	0.959
FM18	55	0.017	0.036	0	1	1	1	0.923	1.000
FM19	343	0.006	0.022	1	0.706	1	13	0.790	0.959
FM20	48	0.211	0.248	0	1	1	4	0.173	0.797
FM21	75	0.238	0.260	0	1	1	4	0.447	0.959
FM22	62	0.407	0.426	0	1	1	2	0.780	0.959
FM23	488	0.203	0.248	1	0.827	1	8	1.000	1.000

MM and FM are male and female modules, respectively. Mod., Module; Gns., Genes; enrich., enrichment; MEG, Memory-Enhancing Gene; MIG, Memory-Impairing Gene. Q-values are FDR-adjusted p-values.

**Fig 3:**
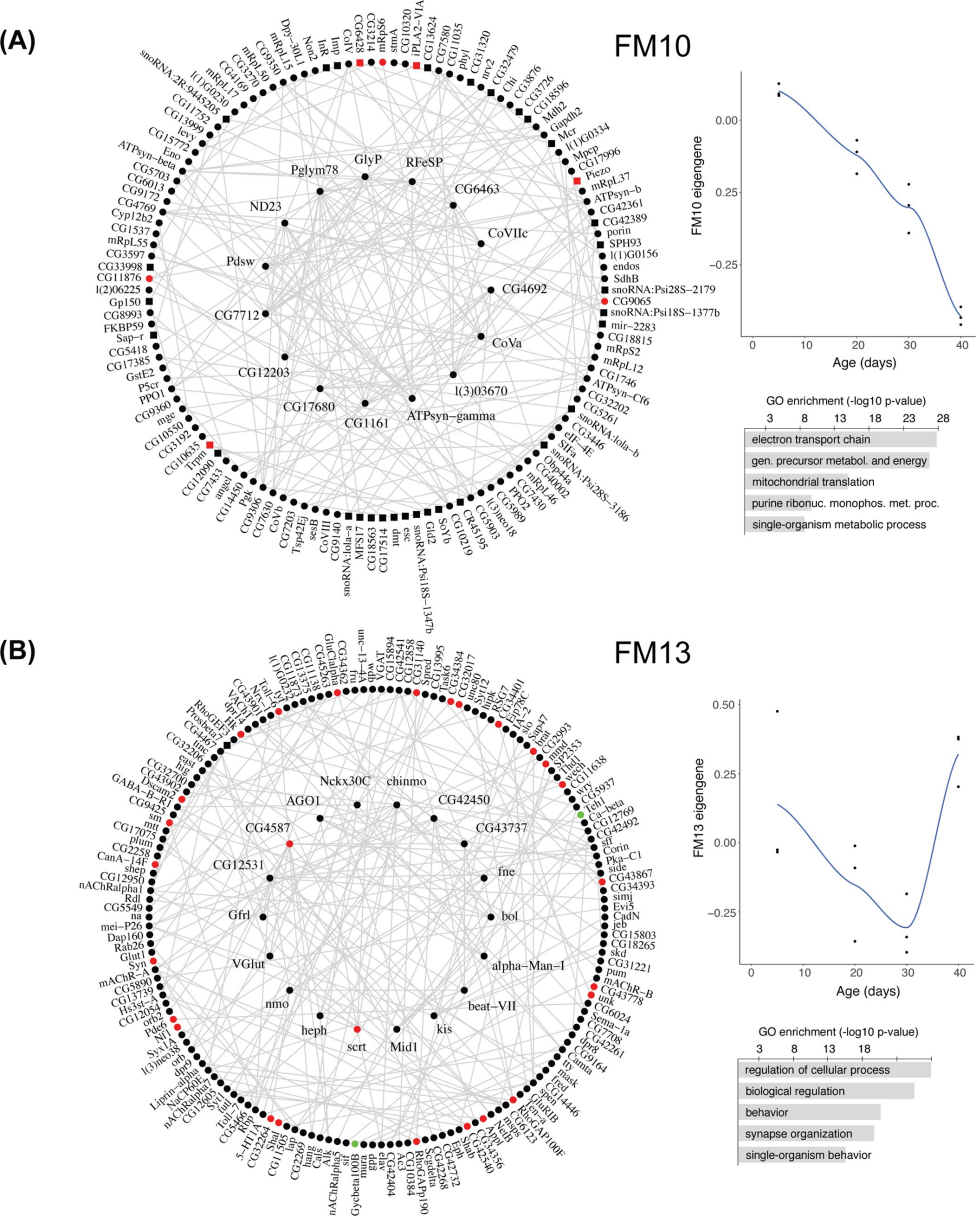
Two of the 23 modules identified in the female dataset by co-expression network analysis. In each panel, the module is plotted on the left (top 200 connections; hub genes are in the center circle); on the right, a LOESS-fitted curve of eigengene values at different ages and the top GO terms are shown (up to 5, listed by decreasing significance; the full lists can be found in S4 Table). (A) FM10, which showed the highest association with age by its eigengene measure. (B) FM13, which had a highly significant enrichment in memory-altering hits from a previous RNAi screen. Genes found through RNAi knockdown to impair memory are colored red; those whose RNAi knockdown enhances memory are colored green. Circles indicate genes positively correlated with the module eigengene; squares are genes negatively correlated with the eigengene. See Table 3 for age-eigengene association and enrichment results for all modules.

Gene ontology enrichment of the modules revealed a variety of functional associations for some of the modules (Fig 3 and S4 Table). In the female data, the module with the strongest effect of age on eigengene variation was FM10 (Table 3 and Fig 3A); its eigengene declined continuously with age, and it was significantly enriched in GO terms related to the electron transport chain, echoing similar findings from the earlier differential expression and temporal clustering analyses. In this module, a large number of genes were anti-correlated to the eigengene, meaning that their expression changes in the opposite direction of the expression of the module as a whole (S5 Table). In any event, the majority of electron transport chain genes within this module were positively correlated with the eigengene, indicating that their expression does decline with age. While the number of modules associated with age (and the overall statistical significance of such associations) was smaller in the male dataset, one of its modules (MM14; S3 Fig) showed a similar decline in expression with age and enrichment in electron transport chain genes. FM10 and MM14 showed significant overlap (p = 4.07 x 10^−68^, hypergeometric probability test).

Another interesting module in the female data was FM13 (Fig 3B). This module, which was also associated with age (Table 3), showed a similar temporal expression pattern as cluster FC15 from the previous analysis (i.e. continuous decline from 5 to 30 days of age, then a steep increase between 30 and 40 days that brings expression back to similar values as in 5-day-old flies; see Fig 2B). Similar to FC15, FM13 also showed a strong enrichment in memory-impairing hits from our published RNAi screen and in GO terms describing neuronal-specific or neuronal-related processes (e.g. “synapse organization” and “cognition”; Fig 3B and S4 Table). The temporal cluster FC15 and the co-expression module FM13 showed very significant overlap (p ≈ 0, hypergeometric probability test).

### Survival study

**Fig 4:**
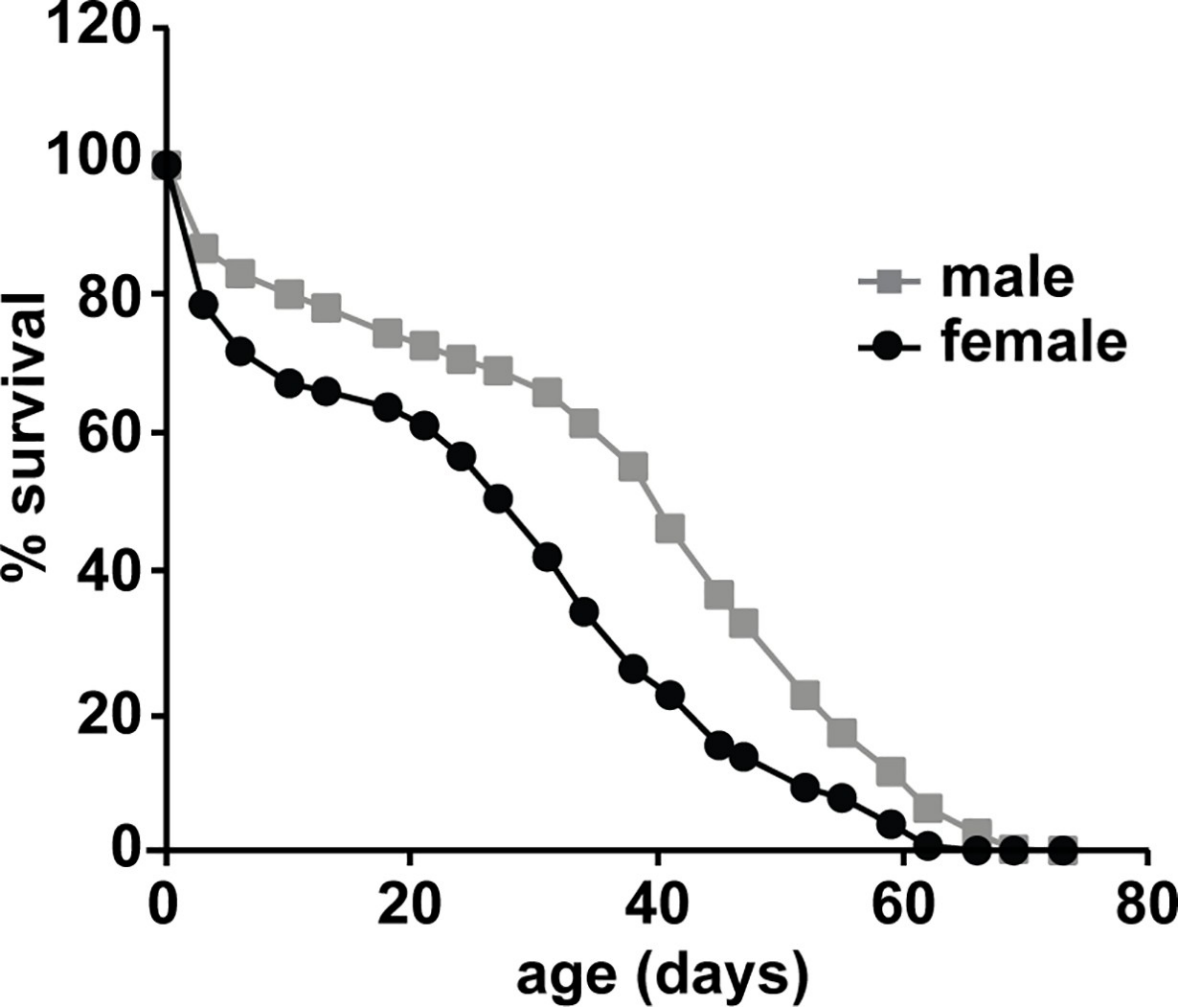
Survival curve for aging flies. 600 CS male and 600 CS female flies were used to generate this graph. The male and female median survival age was 40 and 28 days while the male and female maximum survival age was 69 and 62 days respectively.

We performed a survival analysis to determine the median life expectancy of the fly strain studied here, and to relate survival to the RNA-Seq data (Fig 4). Under our rearing conditions, female flies did not survive as long as male flies. The median survival age (where fifty percent of the flies are still living) was only 28 days for females, whereas for males it was 40 days. However, the maximum survival ages were closer, with females at 62 days of age and males at 69 days of age. Of the four ages tested in our transcriptome and behavior experiments (described below), the oldest group shows the largest sex difference in survival: 50.1% of male flies survived to 40 days of age, whereas only 24.1% of female flies were still living at that age.

### Age-related changes in short and intermediate-term memory performance

**Fig 5:**
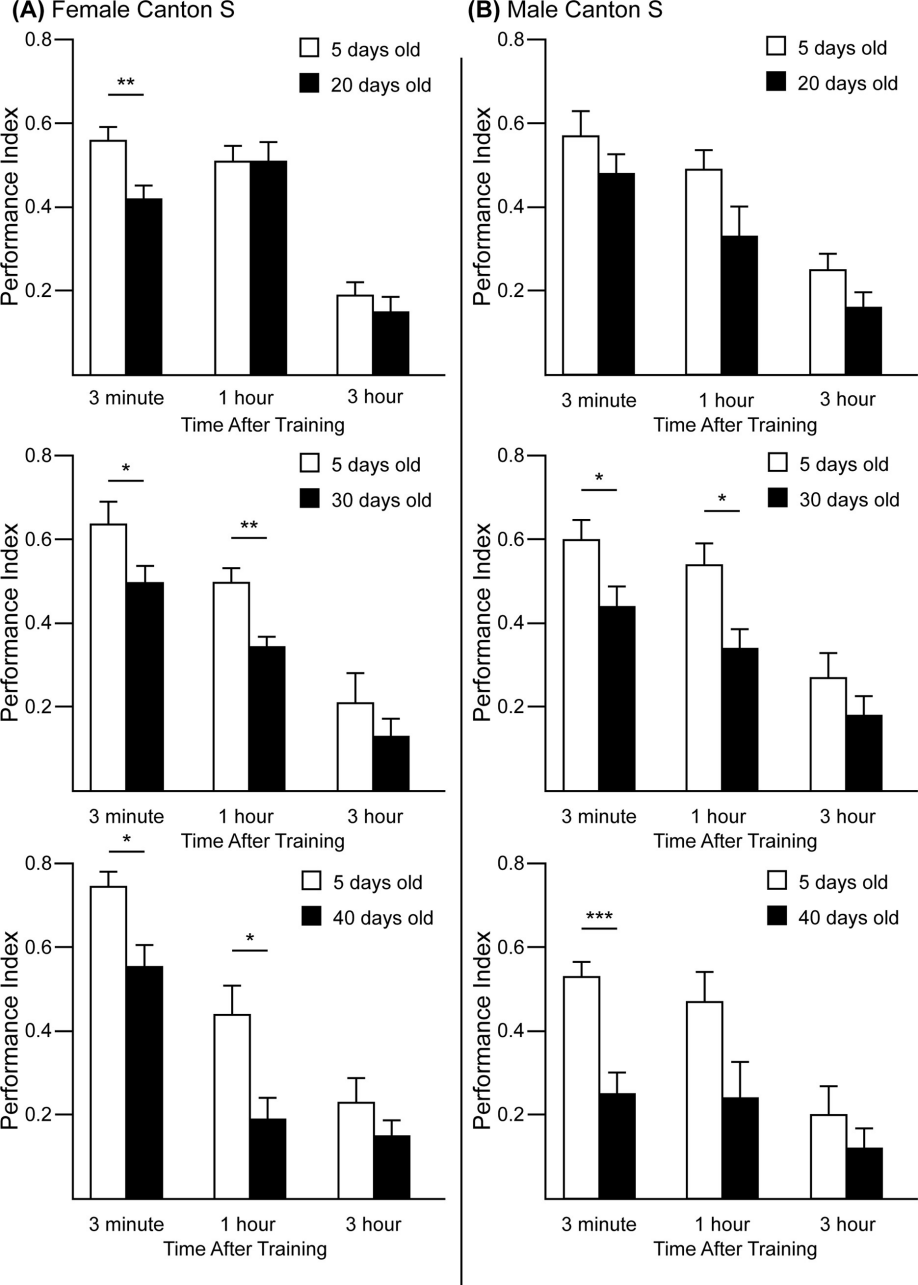
Performance indices measured at multiple times after conditioning CS flies at 20, 30, and 40 days of age compared to control 5-day-old flies. (A) Female CS flies. The Performance Index, tested at 3 min after conditioning, was significantly decreased in 20-, 30- and 40-day-old flies. The Performance Index at 1 hour was significantly decreased in 30- and 40-day-old flies but not at 3 hours, consistent with results of [12]. This is likely due to the reduced sensitivity to extract real differences at low performance values. (B) Male CS flies. The Performance Index was significantly reduced in 30- and 40-day-old flies. The Performance Index at 1 hour was significantly reduced in 30-day-old flies but not at 3 hours.

Increasing memory impairment is a hallmark of the cognitive decline that occurs as part of normal brain aging. In order to relate age-dependent changes in memory performance with transcriptome changes that occur in the brains of the same strain of flies profiled in this study, we compared the Performance Index between young flies and older flies using an olfactory aversive conditioning paradigm (Fig 5). Flies were exposed to two different odors during training, one of which is paired with a series of electric shocks; memory performance was quantified via avoidance of the shock-paired odor in a subsequent two-choice task where each odor was presented at opposite arms of a T-maze. Female flies at 20, 30, and 40 days of age showed a reduced 3-minute Performance Index compared to 5-day-old flies. Male flies showed a similar decline at this time point in the two oldest groups, showing that both sexes have reduced performance with aging. Only 30- and 40-day-old female and 30-day-old male flies showed a significant impairment at 1 hour after conditioning when compared to their respective 5-day-old controls. No significant differences were found in the 3-hour test.

## Discussion

Here we provide whole-brain transcriptome data of the fruit fly *D*. *melanogaster* across several ages and in both sexes. The results from our functional analyses reveal a broad decrease in expression of electron transport chain genes, as well as some increases in expression of immune and stress genes, all of which have been reported in aging studies using brains from other model organisms and humans. We also identify overlapping sets of neuronal function genes that decline in expression between younger and middle-aged flies, but then increase abruptly in expression in the oldest group. Finally, we report age-related deficits in memory test performance, some of which parallel a few of the changes we observed in the transcriptome analyses.

### Downregulation of electron transport chain genes

One of the recurring findings in our study was an age-related decrease in expression of genes from the mitochondrial electron transport chain. Similar findings have been reported in aging transcriptome studies of humans and various other model organisms using brain and other tissues [4]. Mitochondrial function has been deeply linked to the aging process, although cause-effect relationships remain unclear [38, 39]. In certain neurodegenerative disorders—such as Alzheimer’s and Parkinson’s disease—mitochondrial dysfunction is thought to play a role in the early pathophysiology that leads to neuronal death [40]. Somewhat paradoxically, mild reduction in mitochondrial respiratory function, such as through RNAi-mediated knockdown of electron transport chain and other mitochondrial genes, leads to increased lifespans in worms and flies [41, 42], although more severe impairments have the opposite effect in worms and mice [43, 44]. One hypothesis postulates that a retrograde response from the mitochondria to the nucleus, induced by this dysfunction in the electron transport chain, activates the transcription of genes important for stress resistance [45]. In this case, the decrease in expression of electron transport chain genes that occurs with aging might be part of a compensatory mechanism that promotes cell health. In support of this hypothesis, we found an in increase in stress response genes that followed the decrease in expression of mitochondrial genes: in female flies, the GO term “electron transport chain” was enriched among downregulated genes in 20-day-old flies, and the term “response to stress” was enriched among upregulated genes in 30-day-old flies; in male flies, interestingly, the same upregulation of stress response genes only appeared in 40-day-old flies, after the downregulation of electron transport chains that was first detected in 30-day-old flies. Our findings thus provide evidence that the decrease in expression of mitochondrial genes indeed leads to the increase in expression of stress response genes. An alternative explanation for the decreased expression of mitochondrial genes found in older flies is that individual organisms that live longer may already exhibit lowered expression of those genes earlier in life; as they become increasingly overrepresented in older samples, their expression levels are reflected in the pooled transcriptome average. In either case, these reports reinforce the link between mitochondrial health and longevity, which suggests that therapeutic interventions aimed at altering mitochondrial function may be important in slowing the aging process—particularly in energy-demanding tissues such as the brain.

### Decline and reversal of neuronal function gene expression

In the clustering analysis of DE genes, we identified a cluster (FC15) enriched in neuronal and synaptic function genes that displayed a peculiar temporal trajectory: a continuous decline in expression during earlier periods that was sharply reversed in the oldest group. A co-expression module (FM13) with a similar trajectory and GO enrichment was identified in our network analysis of the female dataset, and further testing revealed that FC15 and FM13 overlapped significantly in their gene composition. Some of the hub genes in FM13 have been previously associated with behaviors that are affected by aging. For instance, *Mid1*, a protein found to be associated with sodium leak channels, disrupts locomotor and social clustering behavior when knocked down [46]. Knockdown of *kis*, which encodes a protein involved in chromatin remodeling [47], leads to impairment in locomotor activity and immediate memory recall [48]. Two hub genes, *scrt* and *CG4587*, have also been associated with memory deficits when knocked down in a previous RNAi screen of genes associated with learning and memory [36]; *scrt* is a zinc-finger transcription factor involved in neuronal development [49]; CG4587 encodes a voltage-gated channel subunit that may be important for proper channel localization [50]. In addition to these two hub genes, the FM13 module as a whole was identified in our analysis as being highly enriched in hits from that RNAi screen, particularly in genes that were found to impair memory when repressed in that study. The vast majority of those memory-impairing genes were positively correlated with the module eigengene, indicating that their expression follows the decline in expression of the overall module, and suggesting that they play a role in the progressive deficits in memory that occur during aging.

While the age-related decline in expression of genes involved in cognitive processes makes sense intuitively, the abrupt reversal of this pattern and apparent regain in expression of those genes in the oldest group poses a conundrum. Similar reversals in gene expression have been observed in aging transcriptome of human cortex [51]. It has been shown in mice that gene expression becomes more variable with age, and this may be due to a progressive deterioration of gene repression mechanisms [52, 53]. However, this should cause random genes to have inappropriate increases in expression as a result of aging, in a way that would be highly variable from cell to cell. A more attractive explanation for this concerted reversal pattern of genes with neuronal function is that it might reflect a compensatory upregulation in response to an overall decline in neuronal function. Alternatively, flies that live longer may already express such genes at higher levels earlier in life, and this apparent increase might reflect a higher proportion of such individuals in later samples.

### Sex differences in lifespan

Our survival analysis revealed a shorter lifespan for female flies relative to males. While at least one report using the same strain of flies (Canton-S) found that females lived longer [54, 55], longer male lifespans have been described in other genetic backgrounds [56]. Aside from genetic variation, other factors such as housing, diet, mating status and even odorant exposure are also known to affect lifespan [33, 57, 58]; in some cases, these external factors can impact lifespan to a higher degree in one sex than another. For instance, gut health and microbiota has been shown to play a particularly important role in aging in females, in which dietary interventions can have a larger impact on longevity than it does in males [59]. It is possible that variations in the diet may, at least partly, explain the sex difference in longevity in our study compared to previous reports.

### Age-dependent memory deficits and the transcriptome

Previous studies have found age-related impairments in intermediate- and long-term olfactory memory tests in flies [10–12]. In our experiments, we also observed some age-related deficits in intermediate-term memory when testing flies 1 hour after training. Additionally, we observed an age-dependent deficit in short-term memory when testing flies 3 minutes after training. In females, this deficit was significant in all older groups (20, 30 and 40 days old) compared to the young group (5 days old). In males, the difference was significant only in the two oldest groups (30 and 40 days old). Interestingly, our transcriptome analyses revealed a similar male-delayed pattern for the shift in expression of mitochondrial electron transport chain genes, where a decline was already detectable in 20-day-old females and older, but only in 30-day-old males and older. It is tempting to speculate that some of the deficits in memory performance tests that flies accrue with age are directly associated with decreased mitochondrial function in aging neurons. In our analyses, we did not detect a significant enrichment in memory-impairing genes in any of the clusters and modules that were shown to decline with age and which had an enrichment in energy metabolism GO terms. However, the RNAi screen was performed using young flies [36], which may be more resistant to the effects of mitochondrial dysfunction and associated oxidative stress than older flies. In the future, it would be interesting to test whether improving mitochondrial function in older flies might ameliorate some of the losses in memory performance that develop with aging.

Interestingly, in a recent study where spermidine treatment was shown to suppress age-related memory impairment in flies [20], a transcriptome analysis of treated vs. untreated flies revealed that “oxidation reduction”—one of the most common GO terms among genes affected by aging in our own study—was the top GO term enriched among genes affected by treatment in both 3-day and 10-day-old flies. In that same study, the GO analysis found that terms involved in neuronal function were also enriched among genes affected by spermidine treatment in 10-day-old flies; the effect of spermidine on suppressing age-dependent memory impairment was later shown to be specifically mediated, at least in part, through regulation of the presynaptic active zone size [60]. The GO terms pertaining to the same category (neuronal function and, in particular, synapse organization) were enriched in genes affected by aging in our study. This highlights the value of aging transcriptome studies like ours in uncovering cellular and molecular pathways that are affected by aging, and which may serve as direct targets for investigating therapeutic strategies against age-dependent cognitive impairments.

## Supporting information

S1 Fig Principal component analysis of RNA sequencing data, combining all age groups and both sexes. Samples are labeled according to age and sex. Some separation of the samples according to age is discernible as one moves upward and rightward in the graph. It is notable that variance among the female samples is larger, as observed by the spread across the first component.
(PDF)

S2 Fig Temporal profile of all clusters of DE genes for male and female datasets. Individual lines represent expression of single genes averaged across samples, colored according to the cluster membership values shown in the color bar; only core genes (α ≥ 0.4) are plotted (see Material and Methods).
(PDF)

S3 Fig LOESS-fitted curve of eigengene values for MM14 across all ages. (PDF)

S1 Table List of all DE genes at 5% FDR. (XLSX)

S2 Table GO term enrichment among DE genes. (XLSX)

S3 Table GO term enrichment in temporal clusters. (XLSX)

S4 Table GO term enrichment in co-expression modules. (XLSX)

S5 Table Gene membership values and module assignments. (XLSX)

S1 File Read counts per gene. (TXT)
