# Supplementary figures and images for: Brain transcriptome changes in the aging Drosophila melanogaster accompany olfactory memory performance deficits
Source: PLoS One. 2018 Dec 21;13(12):e0209405. doi: 10.1371/journal.pone.0209405 (PMC6303037; doi:10.1371/journal.pone.0209405)

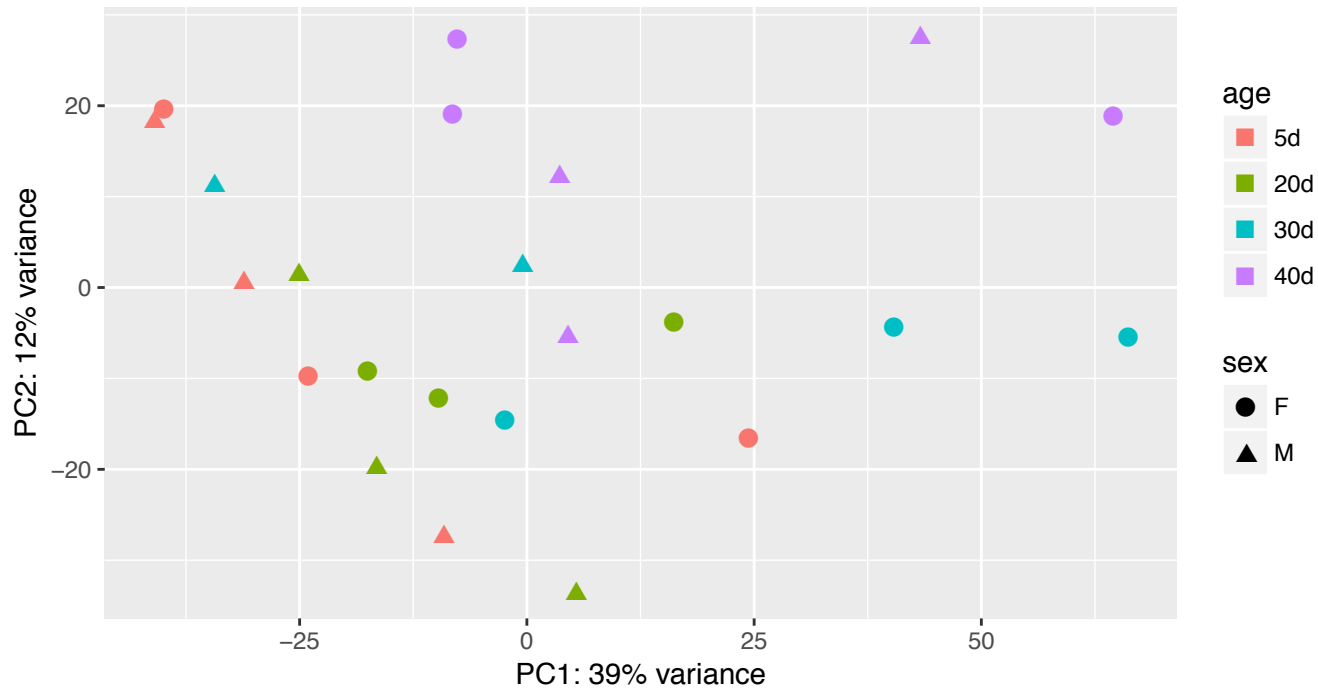

Supplement: S1 Fig — Samples are labeled according to age and sex. Some separation of the samples according to age is discernible as one moves upward and rightward in the graph. It is notable that variance among the female samples is larger, as observed by the spread across the first component. (PDF) [file pone.0209405.s001.pdf]

**Fig. S3**

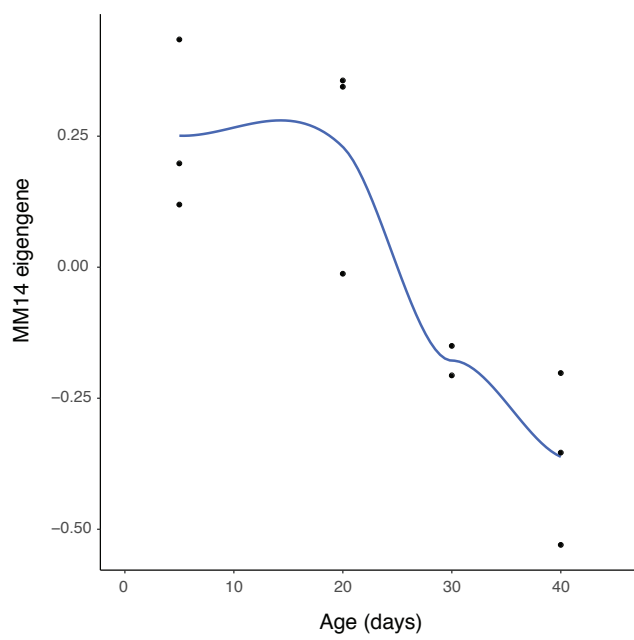

**Supplementary Figure S3** LOESS-fitted curve of eigengene values for MM14 across all ages.

Supplement: S3 Fig — (PDF) [file pone.0209405.s003.pdf]
