# Supplementary material for: Brain transcriptome changes in the aging Drosophila melanogaster accompany olfactory memory performance deficits
Source: PLoS One. 2018 Dec 21;13(12):e0209405. doi: 10.1371/journal.pone.0209405 (PMC6303037; doi:10.1371/journal.pone.0209405)
Supplement: S2 Fig — Individual lines represent expression of single genes averaged across samples, colored according to the cluster membership values shown in the color bar; only core genes (α ≥ 0.4) are plotted (see Material and Methods). (PDF) [file pone.0209405.s002.pdf]

## Fig. S2

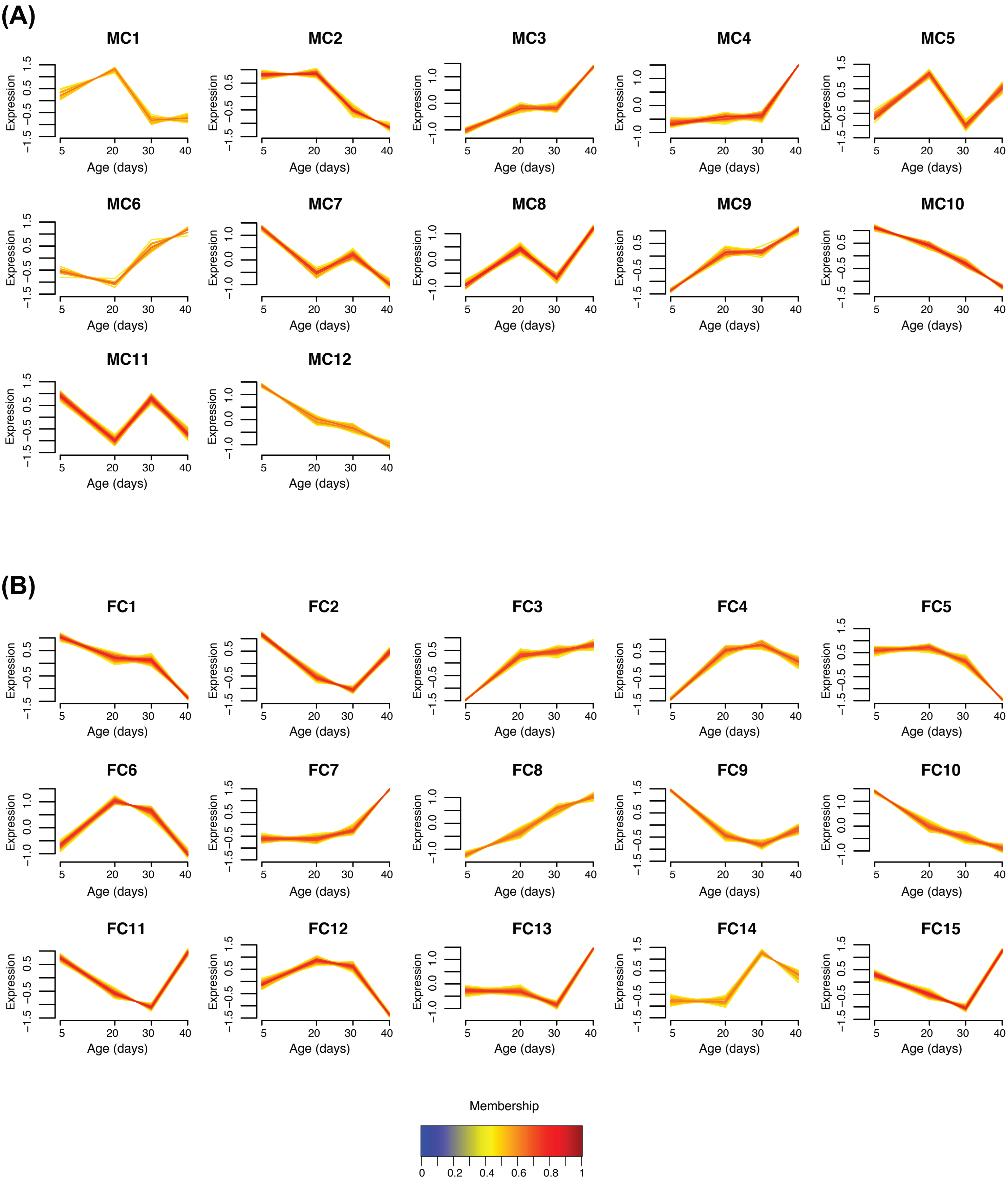


**Supplementary Figure S2** Temporal profile of all clusters of DE genes for male and female datasets. Individual lines represent expression of single genes averaged across samples, colored according to the cluster membership values shown in the color bar; only core genes ($\alpha \geq 0.4$) are plotted (see Material and Methods
